# Professional Advertising

**Published:** 1896-08-08

**Authors:** 


### Professional Advertising.

A good deal of "heat" was evolved in the course of the discussion on "Professional Advertising" in the Ethical Section of the British Medical Association the other day. It was a pity, however, that the amount of "light" was so infinitesimal. The section somewhat resembled a meeting of curates, held to discuss the special grievances of their class. The subject is really important, and it is much to be regretted that it cannot be dealt with in some other way than by an endless repetition of a few stock ideas by the readers of innumerable papers. Will popular orators at annual congresses ever learn that in addition to the outpouring of grievances and the enthusiastic enunciation of obvious popular principles, rational proposals for putting an end to injurious practices are all essential? So far as can be gathered from the reports published, no practical proposal likely to yield fruit was so much as submitted. The case this year seems more hopeless than it did a year ago. In the Ethical Section at King's College in 1895 some definite proposals were made and carried, and a committee, we believe, was appointed to deal thoroughly with the subject. What has become of the committee? Has it, like Rip Van Winkle, gone to sleep, to wake up again about the middle of the next century?

In theory medical men are the most definite persons on earth. The medical lecturer is never tired of urging upon his students the necessity for getting together all the "facts" of a case, and dealing with them and nothing else. But in practice, at any rate, if the Ethical Section of the British Medical Association may be taken as typical of medical business capacity, nobody is so vague and unpractical as the "doctor in council." For example, though hours were spent in discussing the question of medical advertising, nobody even attempted to define what medical advertising might be. Still less did anybody attempt to show wherein its enormity consisted. Probably every doctor in the section would have been willing to admit that it is quite proper for the soapmaker to advertise his soap. But if soap may be advertised, why not medical skill? There are many good reasons why not, but nobody attempted to expound them; nay, more, nobody seemed to be aware that they needed exposition. But these are the very things which need to be done, and to be done again and again. Then we shall all know what is meant when we are asked not to advertise, and we shall know clearly the reason why we are to adopt a rule in life which is entirely different from the ordinary rules that guide the conduct of the commercial man.

The section was told that professional advertising was very shocking and very injurious; and that, *mirabile dictu*, it was indulged in very extensively by some who are "at the head of the profession." Now, either these things are so, or they are not. If they are not so, why this annual attempt at smoking out stinging insects when the insects are not there? But if they are so, is it credible that the whole medical profession in solemn congress assembled cannot devise remedies; cannot even so clearly comprehend the disease as to give it a definite name?

It is a grave thing to say, but there is an impression abroad that the permanent officials of the British Medical Association do not wish to deal in a practical way with this and similar questions. It is hinted that too many persons of "note and name" in connection with the Association have hands which are not so entirely clean as they might be. Of the truth, or otherwise, of this assertion we are not prepared to express an opinion. But of this we feel quite convinced, that if the Association would really take up the question in a practical way, and really make selection from its members of a committee who would take an active and intelligent interest in the subject, and would give such a committee all necessary support, something of a worthy and practical kind would be done within a reasonable period. In the meantime the sorry spectacle is presented of a number of dogs quarrelling over what appears to be a bone, but not one of them is able to say for certain whether it is an actual bone or not.

